# The Superior Response and High Reproducibility of the Memristor-Integrated Low-Power Transparent SnO₂ Gas Sensor

**DOI:** 10.3390/mi15121411

**Published:** 2024-11-23

**Authors:** Taegi Kim, Hee-Dong Kim

**Affiliations:** Department of Semiconductor Systems Engineering, Convergence Engineering for Intelligent Drone, Institute of Semiconductor and System IC, Sejong University, 209, Neungdong-ro, Gwangjin-gu, Seoul 05006, Republic of Korea

**Keywords:** nitric dioxide, SnO_2_ gas sensor, memristor, gasistor

## Abstract

We present a SnO_2_ gas sensor with an HfO_2_ layer that exhibits enhanced performance and reliability for gasistor applications, combining a gas sensor and a memristor. The transparent SnO_2_ gasistor with a 30 nm HfO_2_ layer demonstrated low forming voltages (7.1 V) and a high response rate of 81.28% to 50 ppm of NO_2_ gas, representing an approximately 174.86% increase compared to the response of 29.58% from the SnO_2_ gas sensor without the HfO_2_ layer. The device also showed improved power efficiency and exceptional long-term stability, with reproducibility tests over 10 days at 10 ppm NO_2_ showing a minimal variation of 2.4%. These results indicate that the proposed transparent memristor with the 30 nm HfO_2_ layer significantly enhances the device’s reliability and effectiveness for gasistor applications.

## 1. Introduction

**Figure 1:**
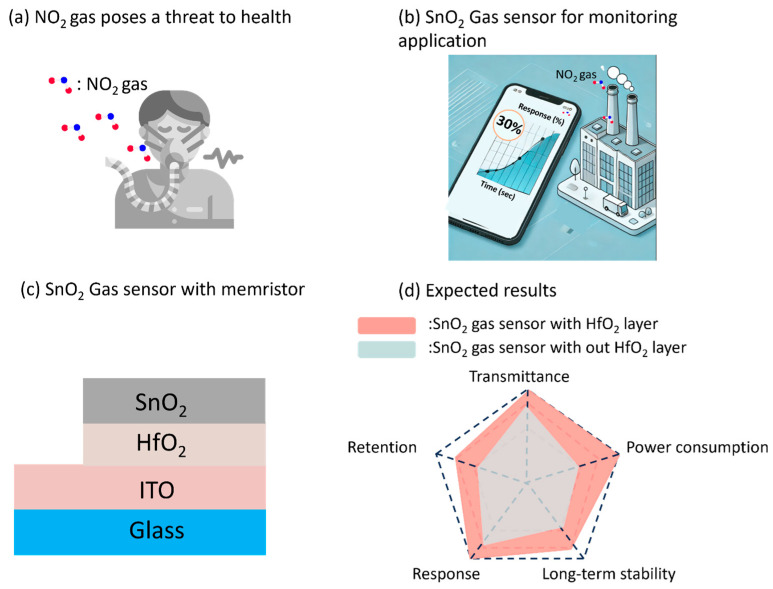
A schematic of a proposed SnO_2_ gasistor. (**a**) Schematic representation of NO_2_, a hazardous gas. (**b**) An overview of efforts to monitor NO_2_ gas. (**c**) A detailed schematic of a transparent SnO_2_ gas sensor with an integrated memristor structure, showing each component: SnO_2_ as the sensing material, HfO_2_ as the insulating layer, and ITO as the transparent conductive electrode. (**d**) A comparison between the SnO_2_ gas sensor and the SnO_2_ gasistor, illustrating the improved performance of the gasistor due to the incorporation of the HfO_2_ layer.

The increasing interest in health monitoring has spurred the development of numerous transparent and wearable devices, including smart devices. Among these advancements, the ability to monitor the concentration of hazardous gases such as NO_2_, CO, C_2_H_6_, etc., is crucial for real-time lung health assessment and disease prevention, as shown in Figure 1a,b. In particular, nitric dioxide (NO_2_) gas sensors have gained considerable attention in the field of medical diagnostics and health monitoring [1,2,3]. The main interest in this area is diagnosing and tracking respiratory diseases such as lung disease, asthma, chronic obstructive pulmonary disease (COPD), and cystic fibrosis [4,5]. In individuals with asthma, when concentrations fall below normal levels, primary ciliary dyskinesia or cystic fibrosis may develop [6,7]. On the other hand, concentrations above normal levels may indicate the need for inhaled corticosteroids or the presence of viral infections. For these reasons, the demand for high-density, cost-effective, selective, and fast-acting gas sensors that can detect trace NO_2_ gas in the air to protect human health and the environment is growing significantly [8,9,10,11]. For instance, Yeom et al. used indium oxide (In_2_O_3_) as a sensing layer to detect NO gas [12]. Similarly, Suehiro et al. used a carbon nanotube gas sensor to detect NO_2_ gas [13]. In particular, SnO_2_ gas sensors have recently gained attention as NO_2_ gas sensors due to their high sensitivity and stability [14,15,16,17]. Alam et al. demonstrated the synthesis and characterization of Cu-SnO_2_ nanoparticles using ultrasonic spray pyrolysis, highlighting the sensitivity and stability of SnO_2_ nanocomposites for gas-sensing applications [18]. Despite these advancements, the requirements for high operating power and low reproducibility remain issues. To address the limitations of conventional gas sensors, we propose a novel SnO₂-based gas sensor integrated with memristor technology, applicable to various gas-sensing applications, as shown in Figure 1c. This study introduces a transparent SnO_2_ gasistor, integrating a memristor structure into a SnO_2_ gas sensor for enhanced performance. Various insulating layers have been utilized in prior studies; however, this work emphasizes the unique benefits of HfO_2_ for gas sensor applications. The HfO_2_ layer in our structure provides superior insulating properties and high thermal stability, effectively suppressing oxygen vacancy migration and stabilizing the CF [19]. This stabilization further enhances the gas-sensing performance by leveraging Joule heating effects, which strengthen the interaction between NO_2_ molecules and the SnO_2_ sensing layer, distinguishing our research from existing SnO_2_-based sensor studies [20]. In this structure, the HfO_2_ layer acts as an insulating material, stabilizing the CF and enhancing the overall gas-sensing performance for NO_2_ detection. As shown in Figure 1d, the introduction of the HfO_2_ layer leads to a more controlled and stable current reduction when exposed to NO_2_ gas, resulting in improved performance, including gas response characteristics. The SnO_2_ layer serves as both the top electrode (TE) and the active gas-sensing material, providing high sensitivity to NO_2_. The inclusion of the HfO_2_ layer significantly stabilizes the gas response while maintaining transparency and low power consumption. Our research explained the operating mechanism of this system, showing how the addition of the HfO_2_ layer boosts the gas-sensing performance. This approach also creates opportunities for expanding the range of potential applications for different target gases, particularly in environmental and health-monitoring systems.

## 2. Materials and Methods

### 2.1. Fabrication of SnO_2_ Gas Sensor with Memristor

Quartz substrates (ITASCO, 16 × 16 × 1 mm^3^) were cleaned by dipping them sequentially in acetone (Sigma-Aldrich, Saint Louis, MO, USA, 99.5%), methanol (Sigma-Aldrich, 99.8%), and deionized water solutions at 60 °C for 10 min each to remove residual impurities. After cleaning, a 100 nm-thick indium tin oxide (ITO) layer was deposited on a quartz substrate using RF magnetron sputtering (Korea Vacuum Tech., Gimpo, Republic of Korea, KVS-2000L) with an ITO target (VTEX, 99.99%) at a power of 100 W. The chamber conditions were maintained at 20 standard cubic centimeters per minute (sccm) of Argon (Ar) flow and 5 millitorrs (mTorr) of pressure. Subsequently, HfO_2_ films with thicknesses of 20, 30, and 40 nm were deposited using RF magnetron sputtering with a HfO_2_ target (ITASCO, 99.99%). Finally, a 100 nm SnO_2_ layer was deposited at room temperature, maintaining the same Ar gas flow rate and pressure as in previous steps. The optical transmittance of the SnO_2_ gasistor with 20, 30, and 40 nm HfO_2_ layers was obtained using a UV–vis spectrophotometer (Cary 5000 UV–vis–NIR spectrophotometer, Agilent Technologies Inc., Santa Clara, CA, USA) with a wavelength accuracy of ±0.1 nm in a spectral range from 300 to 1100 nm.

### 2.2. Electrical and Gas Sensing Characterization of SnO_2_ Gas Sensor with Memristor

The electrical properties were evaluated using a Pulse Generator Unit (Keithley, Cleveland, OH, USA, Keithley 4220) and a Semiconductor Characterization System (Keithley, Cleveland, OH, USA, Keithley 4200). The SnO_2_ was connected to the output terminals of the Keithley 4200 SCS (current: ±0.025%, voltage: ±0.012%) via a triaxial cable using a tailored RF-compatible micro-positioner (MS Tech, Seoul, Republic of Korea, PB50). The bottom electrode (BE) was grounded. The device was placed in a customized aluminum chamber with a volume of approximately 30 cm^3^ to examine the response characteristics of the proposed devices to NO_2_ gas in a controlled setting devoid of any external factors. The chamber was connected to a mass flow controller (MFC; DFPC1000, Daejeon City, Republic of Korea), which in turn was connected to tanks containing NO₂ and high-purity air (99.99%) gases. The NO₂ gas tank used nitrogen (N₂) as an inert gas, with a maximum allowable concentration of 50 parts per million (ppm). Prior to assessing the response characteristics, the humidity in the chamber was eliminated using high-purity air and heating. The temperature and humidity were maintained at a constant level of 25 °C and 20% relative humidity (RH). Before introducing NO_2_ gas, high-purity air was injected into the chamber for 50 s to stabilize it. The NO_2_ concentration was varied to 10, 20, 30, 40, and 50 ppm to examine its influence on the current, while the gas flow rate was set to 500 sccm.

## 3. Results and Discussion

**Figure 2:**
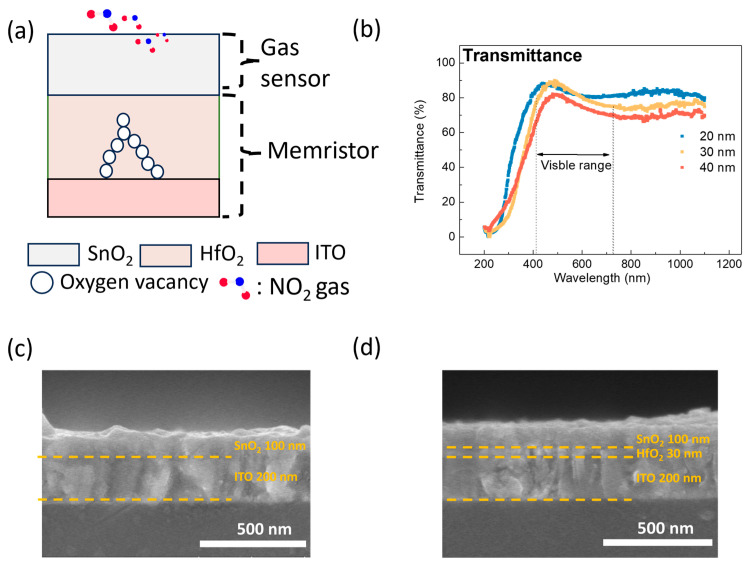
(**a**) A schematic of the SnO2 gasistor structure. (**b**) The transmittance characteristics based on HfO_2_ layer thickness. Cross-sectional images of the SnO_2_ gas sensor: (**c**) with the HfO_2_ layer and (**d**) without the HfO_2_ layer.

Figure 2a illustrates the schematic structure of the proposed transparent SnO₂ gasistor. To achieve transparency, ITO was strategically selected as the bottom electrode (BE) due to its excellent optical transmittance and electrical conductivity, making it ideal for transparent electronic applications [21]. The SnO₂ layer served a dual function, acting as both the top electrode (TE) and the gas-sensing element.

First, to evaluate the transparency of the proposed structures, as illustrated in Figure 2b, the transmittance was measured in the wavelength range of 200 to 1100 nm. As a result, the transmittance in the visible range of 400–700 nm was 85.3%, 85.8%, and 85.9% for HfO_2_ layer thicknesses of 20 nm, 30 nm, and 40 nm, respectively, indicating that the SnO_2_ gasistor is suitable for transparent gas sensor applications [22]. The FE-SEM cross-sectional images in Figure 2c,d provide insight into the structural differences. Figure 2c shows the SnO_2_ gas sensor without the HfO_2_ layer, while Figure 2d depicts the SnO_2_ gasistor with a 30 nm HfO_2_ layer.

**Figure 3:**
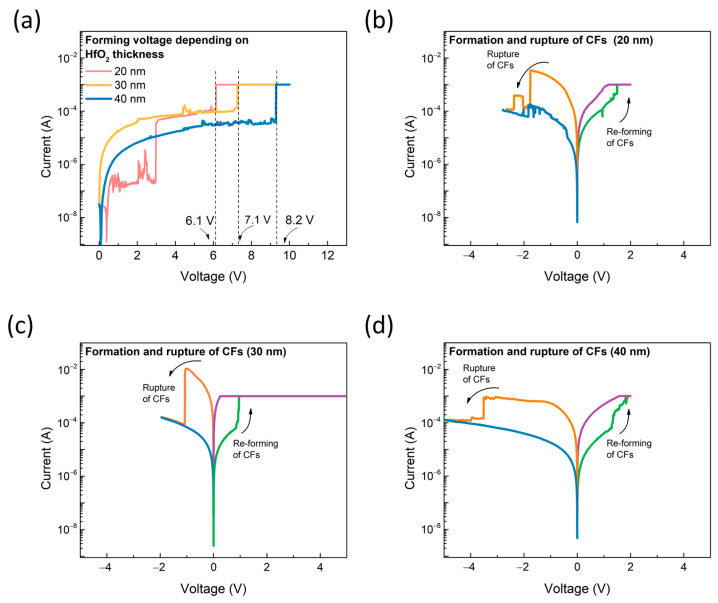
(**a**) The forming process depends on the HfO_2_ thickness. Resistive switching characteristics of the SnO_2_ gasistor with HfO_2_ layer thicknesses of (**b**) 20 nm, (**c**) 30 nm, and (**d**) 40 nm.

The electrical characteristics of the proposed SnO_2_ gasistor were thoroughly examined using a Keithley 4200 parameter analyzer (Keithley, Cleveland, OH, USA, Keithley 4200) to assess its performance under varying conditions. Since the SnO_2_ gasistor operates in a partially broken state of conductive filaments (CFs), the first step was to investigate the formation characteristics of the CFs at different applied voltages. As illustrated in Figure 3a, a forming process was conducted to activate the CFs within the HfO_2_ layer, transitioning the device’s resistance from a high-resistance state (HRS) to a low-resistance state (LRS). This transition is critical for ensuring the proper function of the gasistor, as the CFs are responsible for modulating resistance during gas sensing operations. The forming voltage, required to induce this transition, showed a clear dependence on the thickness of the HfO_2_ layer. Specifically, as the HfO_2_ layer thickness increased, the forming voltage also increased. The measured forming voltages for HfO_2_ layers with thicknesses of 20, 30, and 40 nm were 6.1, 7.1, and 8.2 V, respectively, demonstrating a positive correlation between layer thickness and the required forming voltage. This is due to the thicker HfO_2_ layer increasing the migration distance of oxygen vacancies, necessitating a higher voltage for conductive filament formation. The energy required for oxygen ion movement through the thick insulating layer is higher, thereby enhancing the dielectric properties and resulting in an increased forming voltage [23]. Further investigation into the reset and set characteristics of the device revealed additional insights into its operational behavior. As shown in Figure 3b–d, the reset voltages for the 20, 30, and 40 nm HfO_2_ layers were −1.74 V, −1.08 V, and −3.2 V, respectively, following the forming application. Similarly, the set voltages for these layers were recorded as 1.71 V, 0.96 V, and 2.12 V. Notably, the device with the 30 nm HfO_2_ layer exhibited enhanced structural stability, reflected in the lower required reset and set voltages. This improvement in performance can be attributed to the optimal balance between the filament’s structural stability and the thickness of the HfO_2_ layer, which facilitates more efficient CF formation and disruption. These findings highlight the importance of selecting the appropriate HfO_2_ thickness to achieve optimal device performance, particularly in applications where energy efficiency and stability are critical.

**Figure 4:**
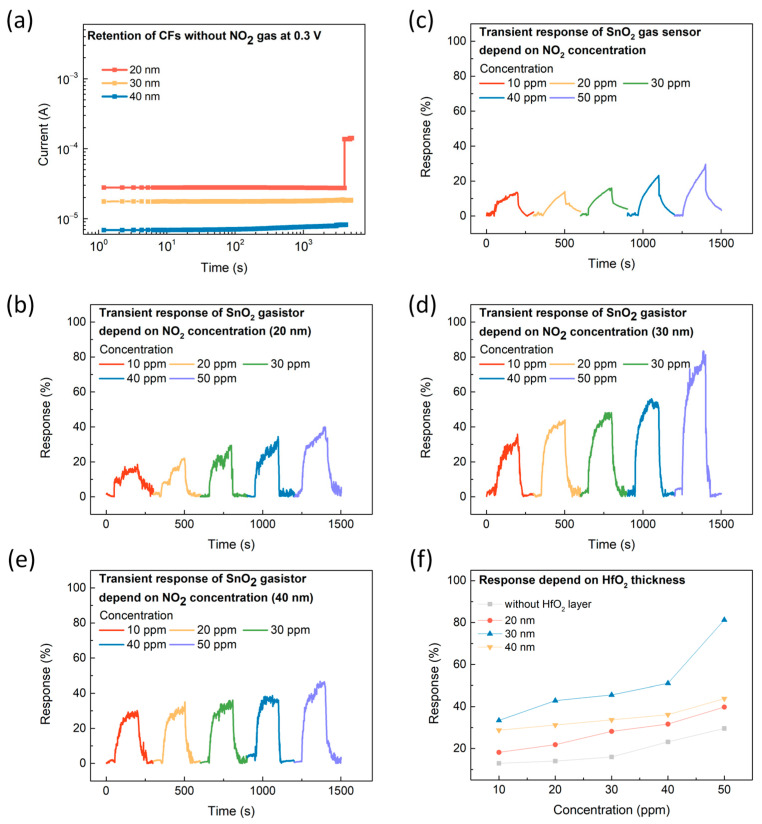
(**a**) Retention characteristics of the CF without NO_2_ gas at 0.3 V for HfO_2_ thicknesses of 20 nm, 30 nm, and 40 nm, measured at room temperature (RT). The transient response of the SnO_2_ gasistor with varying NO_2_ concentrations at 0.3 V and RT (**b**) without an HfO_2_ layer, and at (**c**) 20, (**d**) 30, and (**e**) 40 nm HfO_2_ thicknesses. (**f**) The corresponding response of the SnO_2_ gasistor as a function of HfO_2_ thickness with NO_2_ concentrations ranging from 10 to 50 ppm, measured at RT.

The proposed device operates as a gas sensor in the HRS where the CF is broken. However, previous studies have shown that the resistance state of devices can be converted to an LRS even without gas injection. Thus, we first evaluated the stability of the broken CFs in the absence of gas concerning HfO_2_ thickness. The current was monitored for 4000 s at 0.3 V without gas injection. Figure 4a shows the current value 0.3 V was applied for 4000 s. It was observed that the current remained stable for 4000 s when the HfO_2_ thickness was 30 and 40 nm; however, when the thickness was reduced to 20 nm, the current became unstable. This instability can be attributed to the fact that as the thickness of the HfO_2_ layer decreases, the insulating properties of the material become less effective, leading to increased leakage currents [24,25]. The transient response for each resistance state depending on the HfO_2_ thickness is shown in Figure 4b–e. Gas sensing measurements were conducted in the HRS at RT, ensuring that the CF was in a broken state. This study focused on the NO_2_ sensing characteristics across concentrations ranging from 10 to 50 ppm, chosen to align with standard levels frequently used in gasistor research, thereby facilitating direct comparisons with prior work [26]. At HfO_2_ thicknesses of 20, 30, and 40 nm, a consistent increase in device response was observed as the NO_2_ concentration increased. This proportional relationship suggests that higher gas concentrations enhance the interaction between gas molecules and the sensing material, thereby improving the sensing performance. This proportional relationship indicates that as the gas concentration increases, the interaction between gas molecules and the sensing material is enhanced, leading to improved sensing performance. This phenomenon is attributed to the formation of a Schottky barrier at the SnO_2_-NO_2_ interface, which results in an increase in the overall barrier height [27,28,29]. To accurately compare the changes in electrical conductivity concerning the applied voltage, the response (R) was calculated using the following formula:

(1) $$R=\frac{\left|R_{air}-R_{gas}\right|}{R_{air}}\;\times \;100$$

As depicted in Figure 4f, the SnO_2_ gasistor with a 30 nm HfO_2_ layer demonstrated the highest response characteristics compared to the 20 nm and 40 nm layers. Notably, the SnO_2_ gasistor with a 30 nm HfO_2_ layer demonstrated a response of 81.28% at 50 ppm, compared to 39.77 and 43.81% for the 20 nm and 40 nm devices, respectively. In particular, the SnO_2_ gasistor with a 30 nm HfO_2_ layer demonstrated a 174.86% improvement in response compared to the 29.58% response of the SnO_2_ gas sensor without the HfO_2_ layer, underscoring the superior performance of the 30 nm HfO_2_ layer. This phenomenon can be explained by the optimized thickness of the HfO_2_ layer. In the HRS, the breaking of the CF generates localized heat through the Joule heating effect, which is efficiently transferred to the SnO_2_ sensing layer, enhancing the interaction with NO_2_ molecules [20,26]. The highest gas response observed at a thickness of 30 nm indicates that this thickness provides the best balance between heat generation and insulation properties. The optimized 30 nm thickness maximizes the interaction between the SnO_2_ layer and NO_2_ molecules, promoting stronger adsorption and significantly enhancing sensor performance

**Figure 5:**
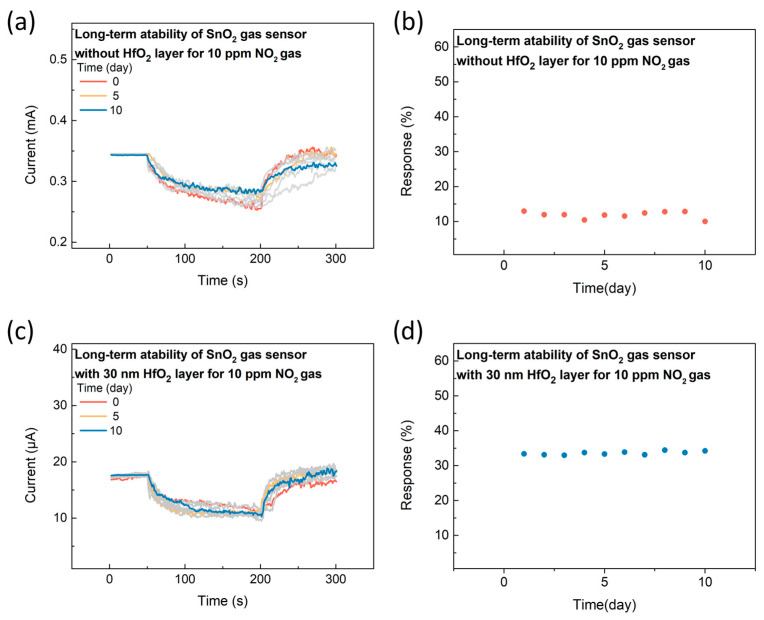
The long-term stability of the SnO_2_ gasistor (**a**) without the HfO_2_ layer and (**c**) with the 30 nm HfO_2_ layer. The corresponding response of (**b**) the SnO_2_ gasistor without the HfO_2_ layer and (**d**) with the 30 nm HfO_2_ layer.

To assess the long-term stability of the SnO_2_ gasistor without the HfO_2_ layer and with a 30 nm HfO_2_ layer, reproducibility measurements were carried out at 25 °C and 20% relative humidity in a sealed chamber, using an applied voltage of 0.3 V and a 10 ppm NO_2_ gas concentration. In comparison to other studies, where gas sensing stability is typically evaluated over a period of 5 to 10 days, our study monitored the transient response of the device over 10 days. As shown in Figure 5a,b, the SnO_2_ gas sensor without the HfO_2_ layer exhibited a high variability of 22.7%. However, as shown in Figure 5c,d, the SnO_2_ gasistor with the 30 nm HfO_2_ layer demonstrated only 2.4% variability in response characteristics, indicating exceptional long-term stability. This low variability strongly suggests the high reproducibility of the gasistor, which is crucial for practical gas sensing applications. The stability is attributed to the effective retention of the CF within the HfO_2_ layer, essential for maintaining reliable gas-sensing performance [29]. The consistent retention of CFs ensures stable operation, preventing degradation of the sensing element and enabling sustained, accurate NO_2_ detection over prolonged periods. This high reproducibility and stability make the SnO_2_ gasistor with the 30 nm HfO_2_ layer a promising candidate for long-term gas sensing in real-environment applications, especially where continuous monitoring is required.

## 4. Discussion

The integration of an HfO_2_ layer into the SnO_2_ gas sensor plays a pivotal role in enhancing its gas-sensing performance and overall stability. The HfO_2_ layer helps stabilize the CF that forms during resistive switching, allowing the sensor to reliably detect low concentrations of NO_2_. By improving the modulation of resistance states, the HfO_2_ layer significantly increases both the sensitivity and reproducibility. Notably, as shown in Table 1, the SnO_2_ gasistor with a 30 nm HfO_2_ layer exhibits the highest performance, achieving a response of 33.39% at 10 ppm, which is considerably superior compared to the 18.17% and 28.73% responses observed with 20 nm and 40 nm layers, respectively. The proposed device demonstrated a recovery time of 87 s for 10 ppm NO_2_ gas, addressing the slow recovery times reported in conventional SnO_2_ gas sensors [30]. Additionally, long-term stability measurements over 10 days revealed only 2.4% variability, indicating significantly higher reproducibility compared to previous studies [31]. These improvements are crucial for ensuring that the sensor operates stably over extended periods, particularly in environments where conditions may fluctuate. This reliability is vital for applications requiring accurate gas detection in real-environmental conditions. Moreover, the incorporation of the HfO_2_ layer reduces power consumption by facilitating efficient CF formation and retention, thereby minimizing energy requirements without compromising performance. The enhanced charge dynamics at the SnO_2_-NO_2_ interface, supported by the HfO_2_ layer, contribute to improved sensing accuracy, especially at lower NO_2_ concentrations. This low-power, high-sensitivity operation makes the SnO_2_ gas sensor with an HfO_2_ layer particularly suitable for transparent, energy-efficient gas-sensing applications, such as environmental monitoring, healthcare diagnostics, and industrial safety systems. In summary, the combination of the SnO_2_ gas sensor and the HfO_2_ layer offers a robust solution for long-term gas sensing. It improves response characteristics, enhances stability, and increases energy efficiency, meeting the growing demands for transparent, low-power electronics and continuous monitoring technologies. This design approach paves the way for advanced applications in environmental and industrial monitoring, providing enhanced performance without sacrificing energy efficiency.

## 5. Conclusions

In this study, we developed a high-performance SnO_2_-based gasistor integrated with a transparent memristor structure, incorporating an HfO_2_ layer for NO_2_ gas sensing applications. The device demonstrated excellent gas-sensing capabilities, particularly with a 30 nm HfO_2_ layer, which significantly improved the response and stability compared to the SnO_2_ gas sensor without the HfO_2_ layer. The gasistor with a 30 nm HfO_2_ layer exhibited a response of 81.28% at 50 ppm of NO_2_ gas, reflecting a 174.86% increase in sensitivity compared to the sensor without the HfO_2_ layer. This improvement is primarily due to the stabilization of CFs within the HfO_2_ layer, ensuring more reliable gas detection and enhanced long-term stability. The device maintained a stable current state for approximately 10,000 s, indicating its potential for extended gas sensing applications. Additionally, the memristor properties of the device amplified charge dynamics at the SnO_2_-NO_2_ interface, modulating the Schottky barrier and allowing the detection of lower NO_2_ concentrations. The SnO_2_ gasistor with a 30 nm HfO_2_ layer also provided an optimal balance between forming voltage and response characteristics, offering superior performance. This approach presents new opportunities for transparent, reliable gas sensors in real-time environmental and health monitoring systems.

## Figures and Tables

**Table 1:** Performance of SnO₂ gasistor with HfO₂ layer of varying thicknesses.

HfO_2_ Thickness (nm)	Forming Voltage (V)	Reset Voltage (V)	Set Voltage (V)	Response at 10 ppm (%)
0	-	-	-	12.96
20	6.1	−1.74	1.71	18.17
30	7.1	−1.08	0.96	33.39
40	8.2	−3.2	2.12	28.73

## Data Availability

The data are contained within the article.

## References

[1] Mamun M.A.A., Yuce M.R. (2020). Recent progress in nanomaterial enabled chemical sensors for wearable environmental monitoring applications. Adv. Funct. Mater..

[2] Yang L., Zheng G., Cao Y., Meng C., Li Y., Ji H., Chen X., Niu G., Yan J., Xue Y. (2022). Moisture-resistant, stretchable NOx gas sensors based on laser-induced graphene for environmental monitoring and breath analysis. Microsyst. Nanoeng..

[3] Yao S., Swetha P., Zhu Y. (2018). Nanomaterial-enabled wearable sensors for healthcare. Adv. Healthc. Mater..

[4] Pijnenburg M., De Jongste J. (2008). Exhaled nitric oxide in childhood asthma: A review. Clin. Exp. Allergy.

[5] Chae M., Lee D., Jung J., Kim H.-D. (2023). Enhanced memristor-based gas sensor for fast detection using a porous carbon nanotube top electrode with membrane. Cell Rep. Phys. Sci..

[6] Walker W., Liew A., Harris A., Cole J., Lucas J. (2013). Upper and lower airway nitric oxide levels in primary ciliary dyskinesia, cystic fibrosis and asthma. Respir. Med..

[7] Lee S., Lee M. (2022). Low-to-moderate atmospheric ozone levels are negatively correlated with hospital visits by asthma patients. Medicine.

[8] Oh E., Choi H.-Y., Jung S.-H., Cho S., Kim J.C., Lee K.-H., Kang S.-W., Kim J., Yun J.-Y., Jeong S.-H. (2009). High-performance NO_2_ gas sensor based on ZnO nanorod grown by ultrasonic irradiation. Sens. Actuators B Chem..

[9] Shendage S., Patil V., Vanalakar S., Patil S., Harale N., Bhosale J., Kim J., Patil P. (2017). Sensitive and selective NO_2_ gas sensor based on WO_3_ nanoplates. Sens. Actuators B Chem..

[10] Sharma B., Sharma A., Myung J.-h. (2021). Selective ppb-level NO_2_ gas sensor based on SnO_2_-boron nitride nanotubes. Sens. Actuators B Chem..

[11] Li Q., Zeng W., Li Y. (2022). Metal oxide gas sensors for detecting NO_2_ in industrial exhaust gas: Recent developments. Sens. Actuators B Chem..

[12] Yeom G., Kwon D., Shin W., Park M.-K., Kim J.-J., Lee J.-H. (2023). Fast-response/recovery In2O3 thin-film transistor-type NO_2_ gas sensor with floating-gate at low temperature. Sens. Actuators B Chem..

[13] Suehiro J., Zhou G., Imakiire H., Ding W., Hara M. (2005). Controlled fabrication of carbon nanotube NO_2_ gas sensor using dielectrophoretic impedance measurement. Sens. Actuators B Chem..

[14] Ding Y., Du B., Guo X., Dong Y., Zhang M., Jin W., Gao C., Peng D., He Y. (2024). An ultrasensitive NO_2_ gas sensor based on a NiO-SnO2 composite with a sub-ppb detection limit at room temperature. Sens. Actuators B Chem..

[15] Park J.H., Kim K.H. (1999). Improvement of long-term stability in SnO_2_-based gas sensor for monitoring offensive odor. Sens. Actuators B Chem..

[16] Mei L., Chen Y., Ma J. (2014). Gas sensing of SnO_2_ nanocrystals revisited: Developing ultra-sensitive sensors for detecting the H2S leakage of biogas. Sci. Rep..

[17] Khuspe G., Sakhare R., Navale S.T., Chougule M.A., Kolekar Y.D., Mulik R.N., Pawar R.C., Lee C., Patil V.B. (2013). Nanostructured SnO_2_ thin films for NO_2_ gas sensing applications. Ceram. Int..

[18] Waqas Alam M., Khatoon U., Qurashi A. (2012). Synthesis and characterization of Cu-SnO_2_ nanoparticles deposited on glass using ultrasonic spray pyrolysis and their H_2_S sensing properties. Curr. Nanosci..

[19] Xu Y.-D., Jiang Y.-P., Tang X.-G., Liu Q.-X., Tang Z., Li W.-H., Guo X.-B., Zhou Y.-C. (2022). Enhancement of Resistive Switching Performance in Hafnium Oxide (HfO_2_) Devices via Sol-Gel Method Stacking Tri-Layer HfO_2_/Al-ZnO/HfO_2_ Structures. Nanomaterials.

[20] Chae M., Lee D., Kim H.D. (2024). Dynamic Response and Swift Recovery of Filament Heater-Integrated Low-Power Transparent CNT Gas Sensor. Adv. Funct. Mater..

[21] Bi Y.G., Liu Y.F., Zhang X.L., Yin D., Wang W.Q., Feng J., Sun H.B. (2019). Ultrathin metal films as the transparent electrode in ITO-free organic optoelectronic devices. Adv. Opt. Mater..

[22] Shah D.K., KC D., Umar A., Algadi H., Akhtar M.S., Yang O.-B. (2022). Influence of efficient thickness of antireflection coating layer of HfO_2_ for crystalline silicon solar cell. Inorganics.

[23] Lee J., Yang K., Kwon J.Y., Kim J.E., Han D.I., Lee D.H., Yoon J.H., Park M.H. (2023). Role of oxygen vacancies in ferroelectric or resistive switching hafnium oxide. Nano Converg..

[24] Kim S., Lee S.-H., Jo I.H., Park T.J., Kim J.H. (2024). Reduced leakage current in atomic-layer-deposited HfO_2_ thin films deposited at low temperature by in-situ defect passivation. Appl. Surf. Sci..

[25] Baik M., Kang H.-K., Kang Y.-S., Jeong K.-S., An Y., Choi S., Kim H., Song J.-D., Cho M.-H. (2017). Electrical properties and thermal stability in stack structure of HfO_2_/Al2O_3_/InSb by atomic layer deposition. Sci. Rep..

[26] Ahmad I., Lee D., Chae M., Kim H.-D. (2024). Advanced recovery and enhanced humidity tolerance of CNTs gas sensor using a filament heater. Chem. Eng. J..

[27] Xing L.-L., He B., Chen Z.-H., Xue X.-Y. (2013). Schottky barrier and catalytic effect induced high gas sensing of one-step synthesized Pd–SnO_2_ nanorods. Solid State Sci..

[28] Zhang B., Zhang S., Xia Y., Yu P., Xu Y., Dong Y., Wei Q., Wang J. (2022). High-performance room-temperature NO2 gas sensor based on Au-loaded SnO2 nanowires under UV light activation. Nanomaterials.

[29] Batzill M. (2006). Surface science studies of gas sensing materials: SnO_2_. sensors.

[30] Leghrib R., Felten A., Pireaux J., Llobet E. (2011). Gas sensors based on doped-CNT/SnO_2_ composites for NO_2_ detection at room temperature. Thin Solid Film..

[31] Xu T., Liu Y., Jiang Z., Pei Y., Zhang S., Xu J., Zhang X., Li X. (2018). Enhancing the NO sensing properties of the SnO_2_ nanowires sensors by Ar–O_2_ plasma modification. J. Mater. Sci. Mater. Electron..

